# Comparison of the Value of Four Objective Nutritional Indices in Assessing the Long-Term Prognosis of Elderly Patients with Heart Failure with Preserved Ejection Fraction

**DOI:** 10.31083/j.rcm2506201

**Published:** 2024-05-31

**Authors:** Xingman Fan, Qiongyi He, Kaijie Zhang, Xiaohua Lan, Yanyan Li, Haitao Zhang

**Affiliations:** ^1^Graduate School, Hebei North University, 075000 Zhangjiakou, Hebei, China; ^2^Air Force Clinical Medical College, The Fifth School of Clinical Medicine, Anhui Medical University, 230032 Hefei, Anhui, China; ^3^Department of Cardiology, Air Force Medical Center, Air Force Medical University, PLA, 100142 Beijing, China

**Keywords:** heart failure with preserved ejection fraction (HFpEF), prognosis, objective nutrient score, older

## Abstract

**Background:**

The long-term prognosis of heart failure with preserved ejection fraction (HFpEF) is influenced by malnutrition. Currently, there’s a deficit in objective and comprehensive nutritional assessment methods to evaluate the nutritional status and predicting the long-term outcomes of HFpEF patients.

**Methods:**

Our retrospective study included two hundred and eighteen elderly HFpEF patients admitted to the cardiovascular ward at the Air Force Medical Centre from January 2016 to December 2021. Based on follow-up outcomes, patients were categorized into all-cause death (99 cases) and Survival (119 cases) groups. We compared general data, laboratory results, and nutritional indexes between groups. Differences in subgroups based on Triglyceride-Total Cholesterol-Body Weight Index (TCBI), Geriatric Nutritional Risk Index (GNRI), Prognostic Nutritional Index (PNI), and Controlled Nutrition Score (CONUT) were analyzed using Kaplan-Meier survival curves and log-rank test. COX regression was used to identify all-cause mortality risk factors, and the predictive accuracy of the four nutritional indices was assessed using receiver operating characteristic (ROC) curves and Delong test analysis.

**Results:**

A total of 101 (45.41%) HFpEF patients experienced all-cause mortality during 59.02 ± 1.79 months of follow-up. The all-cause mortality group exhibited lower GNRI and PNI levels, and higher CONUT levels than the Survival group (*p* < 0.05). Kaplan-Meier analysis revealed lower cumulative survival in the low GNRI (≤96.50) and low PNI (≤43.75) groups, but higher in the low CONUT (≤2) group, compared to their respective medium and high-value groups. Multifactorial COX regression identified low PNI (≤43.75) as an independent all-cause mortality risk factor in elderly HFpEF patients. ROC and Delong’s test indicated PNI (area under the curve [AUC] = 0.698, 95% confidence interval [CI] 0.629–0.768) as a more effective predictor of all-cause mortality than TCBI (AUC = 0.533, 95% CI 0.456–0.610) and CONUT (AUC = 0.621, 95% CI 0.547–0.695; *p* < 0.05). However, there was no significant difference compared to GNRI (AUC = 0.663, 95% CI 0.590–0.735; *p* > 0.05).

**Conclusions:**

In elderly HFpEF patients a PNI ≤43.75 was identified as an independent risk factor for all-cause mortality. Moreover, PNI demonstrates superior prognostic performance in predicting all-cause mortality in elderly patients with HFpEF when compared to TCBI, GNRI, and COUNT.

## 1. Introduction

Heart failure with preserved ejection fraction, or HFpEF, has emerged as a major subtype of heart failure (HF), affecting up to 3 million people in the United States alone [1]. Due to the pathophysiological heterogeneity of the disease, conventional pharmacological treatments such as diuretics, reduce congestion and edema without significantly improving HFpEF prognosis, which boasts a 50% 5-year survival rate [2]. As a result, our objective is to identify effective and affordable prognostic markers for HFpEF patients.

A significant proportion of hospitalized HF patients (34–70%) experience malnutrition, a factor that is often overlooked in standard risk assessment. Recent studies have demonstrated [3, 4] that malnutrition is closely linked to the incidence of a poor prognosis in HFpEF patients. In response, novel nutritional indices have been developed to integrate objectively measurable parameters including body weight, total cholesterol, triglycerides, and additional variables for a comprehensive and objective assessment. Notable among these are the Geriatric Nutritional Risk Index (GNRI), Prognostic Nutritional Index (PNI), Controlled Nutrition Score (CONUT), and Triglyceride-Total Cholesterol-Body Weight Index (TCBI). While these indices are widely used in nutritional evaluation of patients with cancer [5] and other cardiovascular disorders [6], their application to HFpEF prognosis remains underexplored. Our study aims to fill this gap by evaluating these nutritional screening indices in elderly HFpEF patients, analyzing their relationship with long-term prognosis and assessing both their advantages and limitations.

## 2. Methods

### 2.1 Study Population

**Fig. 1.**
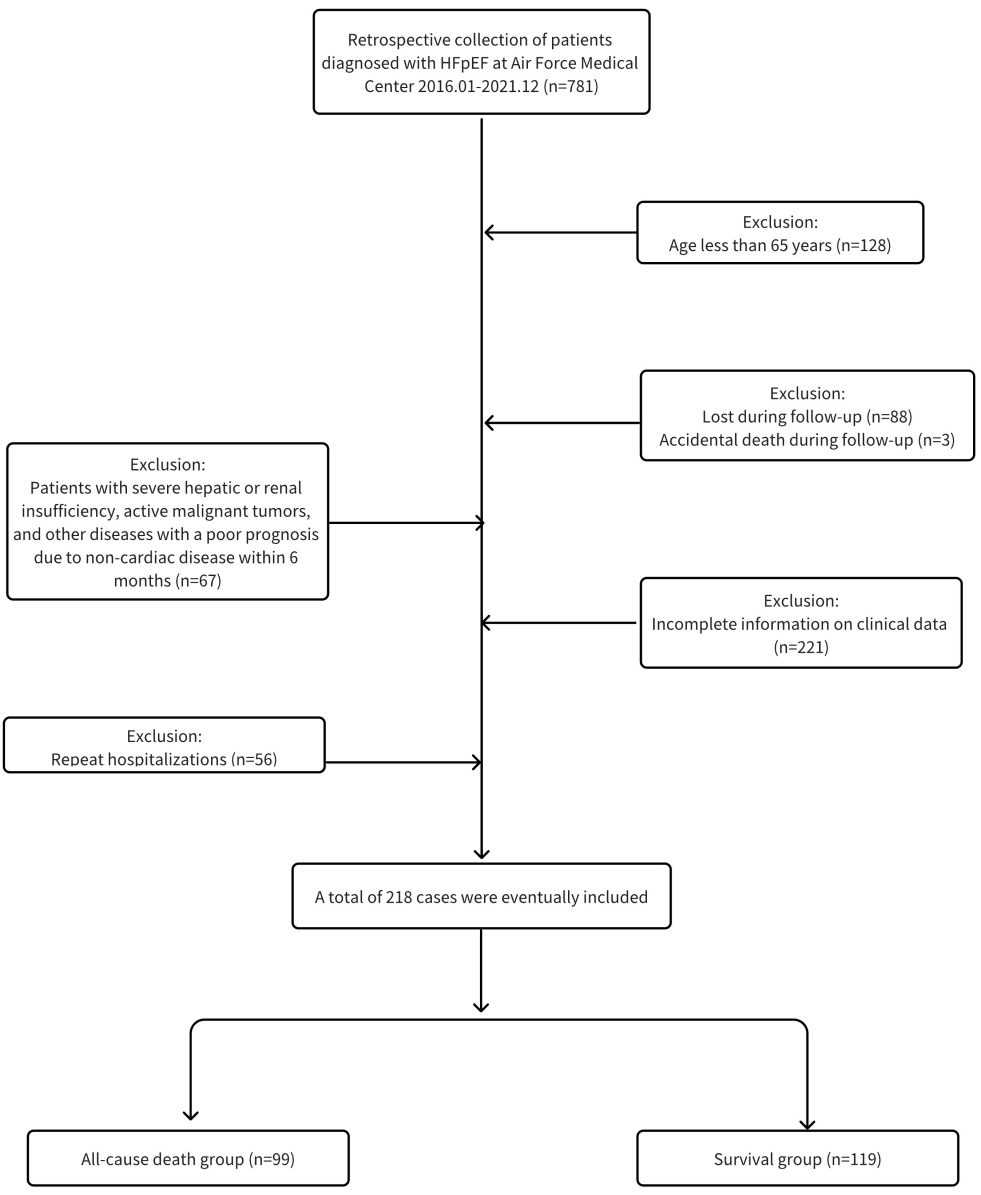
**Flow diagram indicating study population.** HFpEF, heart failure with preserved ejection fraction.

This is a single-center, retrospective cohort study involving 781 patients with HFpEF who were hospitalized in the cardiovascular medicine cadre ward at the Air Force Medical Centre from January 2016 to December 2021. After applying inclusion and exclusion criteria (refer to Fig. 1 for the specific flow chart), 218 patients were ultimately included, comprising 147 males and 71 females, with an average age of 85 years (range: 77–89). Among them, 52 individuals (23.85%) experienced the decompensated period of heart failure with preserved ejection fraction. The inclusion criteria were as follows: (1) age ≥65 years; (2) patients with HFpEF meeting the diagnostic criteria of Chinese Heart Failure Diagnosis and Treatment Guidelines 2018 [7]; and (3) complete clinical data. Exclusion criteria included: (1) Patients with severe hepatic insufficiency, active malignancy and other diseases with poor prognosis within 6 months due to non-cardiac diseases; (2) patients with severe aortic stenosis, aortic regurgitation, mitral stenosis or mitral regurgitation caused by valve structural changes found by transthoracic echocardiography at admission; and (3) patients who died due to accidents during follow-up or were lost during follow-up.

Since this study is retrospective, it received an exemption from the ethics committee of the Air Force Medical Center of the Chinese People’s Liberation Army, and the requirement of informed consent was waived. All research methods were in accordance with the Declaration of Helsinki and relevant guidelines/regulations.

### 2.2 Research Methods

#### 2.2.1 Information Collection

The electronic medical record system was reviewed to collect baseline data during the first hospitalization. This included age, sex, body mass index (BMI), New York heart association (NYHA) classification, systolic and diastolic blood pressure, heart rate, and comorbidities such as hypertension, atrial fibrillation, diabetes mellitus, chronic kidney disease, chronic obstructive pulmonary disease, hyperlipidemia, and hyperuricemia. Medications taken upon admission, such as angiotensin-converting enzyme inhibitor (ACEI), angiotensin receptor blocker (ARB), angiotensin receptor neprilysin inhibitors (ARNI), beta-blockers, calcium channel blockers (CCBs), digoxin, statins, intravenous diuretics, hydrocorticosteroid antagonists, and antiplatelet drugs were also recorded. Laboratory markers within 24 hours of admission, including hemoglobin (Hb), lymphocyte count (ALC), B-type natriuretic peptide (BNP), sodium (Na), potassium (K), creatinine (CCR), blood urea nitrogen (BUN), estimated glomerular filtration rate (eGFR), high-density lipoprotein (HDL), low-density lipoprotein (LDL), total cholesterol (TC), triglycerides (TG), prealbumin (PA), albumin (ALB), total protein (TP), D-dimer, were documented. Additionally, left ventricular ejection fraction (LVEF), and nutritional indices such as TCBI, GNRI, CONUT, and PNI were calculated using their respective formulas.

#### 2.2.2 TCBI, GNRI, PNI Definitions and Subgroups

Calculations were performed as follows. (1) TCBI = TG (mg/dL) × TC (mg/dL) × body weight (kg)/1000. Patients were divided into low (<756.57), medium (756.57–1251.49), and high TCBI groups (>1251.49) based on TCBI tertiles. (2) PNI = ALB (g/L) + 5 × lymphocyte count (× 109). Patients were divided into low (<43.75), medium (43.75–48.40), and high PNI groups (>48.40) based on PNI tertiles. (3) GNRI = 14.89 × ALB (g/dL) + 41.7 × (body weight (kg)/ideal body weight (kg)). If the patient’s weight exceeds the ideal weight, the ratio of weight to ideal weight is set as 1 [8]. The ideal weight for men = height (cm) – 100 – ((height (cm) – 150)/4), the ideal weight for women = height – 100 – ((height – 150)/2.5). Patients were divided into low (<96.50), medium (96.50–102.45), and high GNRI groups (>102.45) based on GNRI tertiles.

#### 2.2.3 CONUT Definitions and Subgroups

CONUT, a commonly used nutritional screening tool for hospitalized patients [9], was obtained by cumulative summation of serum albumin, total cholesterol, and lymphocyte count scores. A score of ALB ≥35 g/L, 30–34.9 g/L, 25–29.9 g/L, and ≤24.9 g/L corresponds to 0, 2, 4, and 6 points, respectively. Lymphocyte counts ≥1.6 × ${\mathrm{10}}^{9}$/L, 1.2–1.59 × ${\mathrm{10}}^{9}$/L, 0.8 × ${\mathrm{10}}^{9}$ to 1.19 × ${\mathrm{10}}^{9}$/L, and <0.8 × ${\mathrm{10}}^{9}$/L are scored as 0, 1, 2, and 3 points, respectively. Similarly, TC ≥180 mg/dL, 140 to 179 mg/dL, 100 to 139 mg/dL, and ≤99 mg/dL correspond to 0, 1, 2, and 3 points, respectively. A higher total score indicates a poorer nutritional status. Patients are then classified into three groups based on CONUT tertiles: low CONUT group (≤2), medium CONUT group (2–4), and high CONUT group (>4).

#### 2.2.4 Follow-up and Endpoint Events

The first day of discharge was taken as the starting time point of follow-ups. They were conducted through electronic medical record review, outpatient visits, and telephone consultations. This included monitoring for the occurrence of main outcome events and noting the time of their occurrence. The primary outcome event under observation was all-cause death. The follow-up period extended until June 1, 2023.

#### 2.2.5 Statistical Analysis

Data analysis was conducted using SPSS 26.0 (IBM Corp, Armonk, NY, USA). Quantitative data with normal distribution were expressed as mean ± standard deviation ($\bar{X}$ ± S) and compared between groups using independent samples *t*-test. Quantitative data with skewed distribution were expressed as median [M (P25, P75)] and compared between groups using the Mann-Whitney U rank-sum test. Counting data were expressed as cases (%) and compared between groups using χ^2^ or Fisher’s exact probability method. The Kaplan-Meier method was used to plot survival curves, and a comparison between groups was done using the log-rank test. Univariate and multivariate COX regressions were used to analyze the long-term prognostic effects of TCBI, GNRI, PNI, and CONUT on elderly patients with HFpEF. To assess the predictive value of the four objective nutritional indices on all-cause mortality in elderly patients with HFpEF, ROC curves were generated, and the area under the curve (AUC) was analyzed. Differences in AUC were compared using the DeLong test. Statistical significance was set at *p* < 0.05.

## 3. Results

### 3.1 Analysis of Baseline Data

Our study enrolled a total of 218 patients with heart failure and preserved ejection fraction. These patients were categorized based on follow-up outcomes as either all-cause mortality or survival. When compared to the Survival group, individuals in the all-cause mortality group were older and exhibited a higher prevalence of chronic kidney disease. Furthermore, this group showed elevated levels of BNP, K, CCR, BUN, and CONUT (*p* < 0.05), along with decreased levels of BMI, LVEF, statin, antiplatelet medication, ACEI/ARB/ARNI utilization, lymphocyte count, Hb, Na, eGFR, ALB, GNRI, and PNI (*p* < 0.05). The remaining indicators did not exhibit statistically significant differences (*p* > 0.05), as illustrated in Table 1.

**Table 1.** **Comparison of clinical data between groups with all-cause mortality and Survival group**.

Variation		All-cause mortality (n = 99)	Survival group (n = 119)	Statistics	*p*-value
Age (year)		87.00 (82.00, 91.00)	82.00 (74.00, 87.00)	–4.981	0.001
BMI (kg/$m^{2}$)		22.82 ± 3.90	24.99 ± 3.45	4.358	0.001
SBP (mmHg)		143.00 (122.00, 158.00)	136.00 (126.00, 155.00)	–0.016	0.987
DBP (mmHg)		72.00 (66.00, 80.00)	71.00 (65.00, 82.00)	–0.338	0.736
Heart rate (bpm)		72.00 (68.00, 86.00)	76.00 (66.00, 86.00)	–0.155	0.877
LVEF (%)		56.00 (54.00, 58.00)	57.00 (54.00, 59.00)	–2.346	0.019
Male (n (%))		72 (72.7%)	75 (63.0%)	2.316	0.128
NYHA (n (%))				5.344	0.148
	I	12 (12.1%)	18 (15.1%)		
	II	36 (36.4%)	43 (36.1%)		
	III	35 (35.4%)	50 (42.0%)		
	IV	16 (16.2%)	8 (6.7%)		
Comorbidities (n (%))					
	Hypertension	83 (83.8%)	97 (81.5%)	0.203	0.652
	Atrial fibrillation	26 (26.3%)	44 (37.0%)	2.845	0.092
	Diabetes	32 (32.3%)	39 (32.8%)	0.005	0.944
	Chronic renal disease	38 (38.4%)	23 (19.3%)	9.738	0.002
	Chronic obstructive pulmonary Disease	15 (15.2%)	14 (11.8%)	0.537	0.463
	Hyperuricemia	15 (15.2%)	11 (9.2%)	1.796	0.180
	Hypertriglyceridemia	29 (29.3%)	44 (37.0%)	1.432	0.231
Medications (n (%))					
	ACEI/ARB/ARNI	25 (25.3%)	50 (42%)	6.730	0.009
	Beta blockers	47 (47.5%)	70 (58.8%)	2.799	0.094
	CCB	44 (44.4%)	53 (44.5%)	0.001	0.989
	Digoxin	6 (6.1%)	12 (10.1%)	1.155	0.283
	Statins	45 (45.5%)	91 (76.5%)	22.154	0.001
	Intravenous diuretics	39 (32.8%)	36 (36.4%)	0.309	0.578
	Hydrocorticosteroid antagonist	42 (42.4%)	51 (42.9%)	0.004	0.949
	Antiplatelet drug	46 (46.5%)	76 (63.9%)	6.640	0.010
Laboratory experiment					
	BNP (pg/mL)	255.00 (109.43, 814.00)	182.30 (77.70, 327.00)	–2.740	0.006
	ALC (${\mathrm{10}}^{9}$/L)	1.05 (0.80, 1.50)	1.40 (0.98, 1.92)	–3.989	0.001
	HB (g/L)	112.00 (96.00, 126.00)	124.00 (109.00, 134.00)	–3.450	0.001
	Na (mmol/L)	139.00 (136.00, 141.00)	139.00 (137.00, 142.00)	–2.073	0.038
	K (mmol/L)	4.20 (3.90, 4.60)	4.10 (3.80, 4.30)	–2.708	0.007
	CCR (µmol/L)	114.00 (83.00, 211.00)	82.00 (64.00, 116.00)	–4.050	0.001
	BUN (mmol/L)	9.60 (6.90, 14.40)	7.30 (5.50, 9.00)	–4.186	0.001
	e GFR (mL·min·(1.73 $m^{2}$)^-1^)	57.25 (27.00, 84.87)	85.45 (56.31, 116.50)	–3.853	0.001
	HDL (mmol/L)	1.05 (0.88, 1.30)	1.04 (0.89, 1.28)	–0.203	0.839
	LDL (mg/dL)	2.27 (1.71, 2.80)	2.09 (1.59, 2.66)	–1.322	0.186
	TG (mmol/L)	1.13 (0.80, 1.60)	1.19 (0.90, 1.63)	–0.950	0.342
	TC (mmol/L)	3.99 (3.28, 4.77)	3.75 (3.09, 4.32)	–1.716	0.086
	PA (mg/L)	195.66 ± 64.90	211.92 ± 61.00	1.903	0.058
	ALB (g/L)	38.71 ± 3.91	40.31 ± 3.52	3.172	0.001
	TP (g/L)	65.62 ± 7.01	65.95 ± 5.47	0.382	0.703
Objective nutritional indicators					
	TCBI	918.18 (648.83, 1417.76)	1011.17 (624.97, 1509.99)	–0.831	0.406
	GNRI	97.24 (93.37, 102.15)	101.41 (97.09, 104.83)	–4.134	0.001
	PNI	44.36 ± 4.64	47.78 ± 4.76	5.341	0.001
	CONUT	3.42 ± 1.49	2.78 ± 1.34	–3.355	0.001

Abbreviations: BMI, body mass index; SBP, systolic blood pressure; DBP, diastolic blood pressure; LVEF, left ventricular ejection fraction; NYHA, New York heart association; ACEI, angiotensin-converting enzyme inhibitor; ARB, angiotensin receptor blocker; ARNI, angiotensin receptor neprilysin inhibitors; CCB, calcium channel Blockers; BNP, b-type natriuretic peptide; ALC, lymphocyte count; HB, hemoglobin; Na, sodium; K, potassium; CCR, creatinine; BUN, blood urea nitrogen; e GFR, estimate glomerular filtration rate; HDL, high-density lipoprotein; LDL, low-density lipoprotein; TG, triglyceride; TC, total cholesterol; PA, prealbumin; ALB, albumin; TP, total protein; TCBI, Triglyceride-Total Cholesterol-Body Weight Index; GNRI, Geriatric Nutritional Risk Index; PNI, Prognostic Nutritional Index; CONUT, Controlled Nutrition Score; n, number.

### 3.2 Comparison of the Percentage of Patients in the All-Cause Dead Group and the Distant All-Cause Mortality Rate for Subgroups with Different Objective Nutritional Indicator Levels

**Fig. 2.**
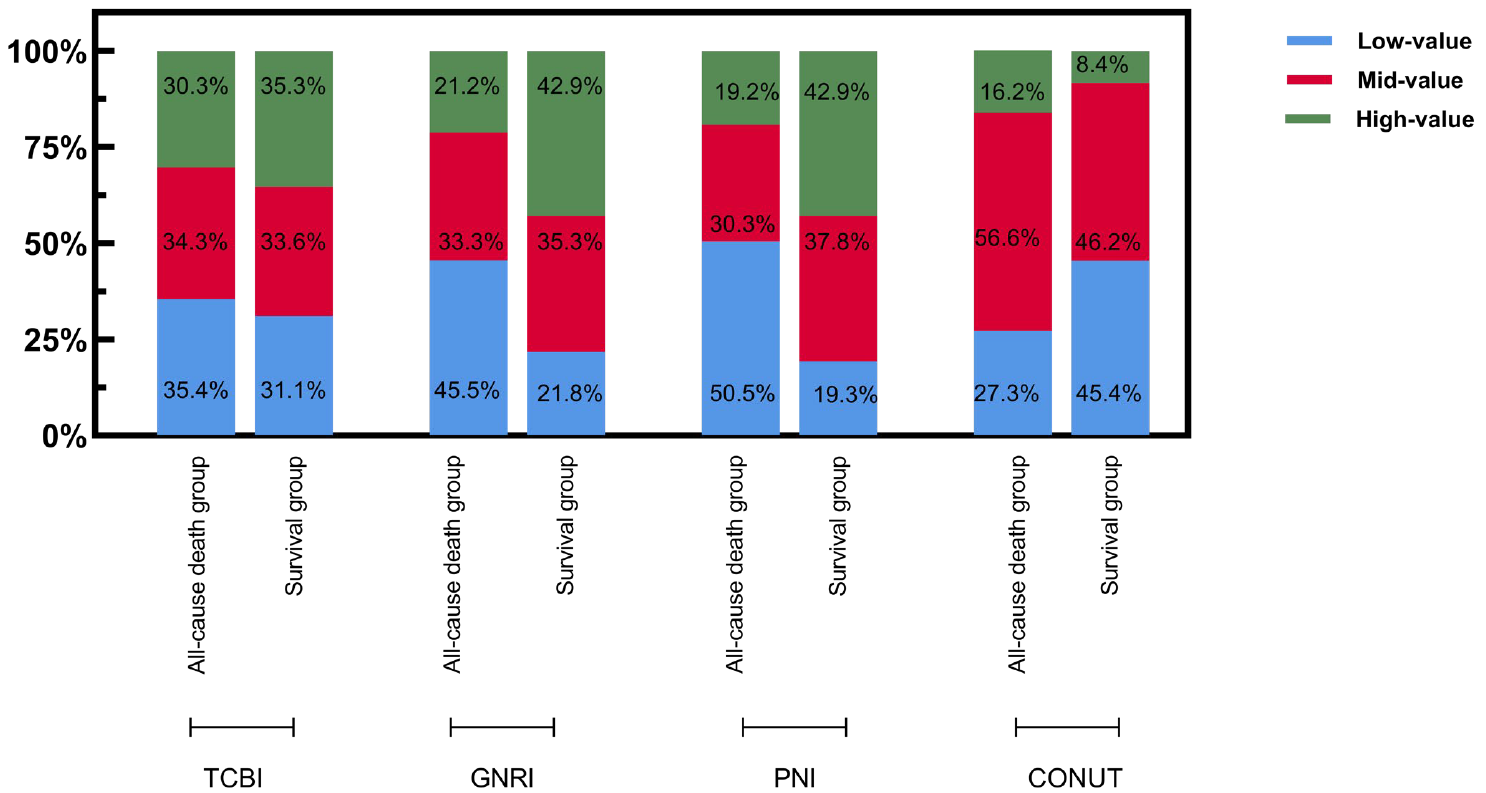
**Grouping distribution of four objective nutritional indicators.** Abbreviation: TCBI, Triglyceride-Total Cholesterol-Body Weight Index; GNRI, Geriatric Nutritional Risk Index; PNI, Prognostic Nutritional Index; CONUT, Controlled Nutrition Score.

**Fig. 3.**
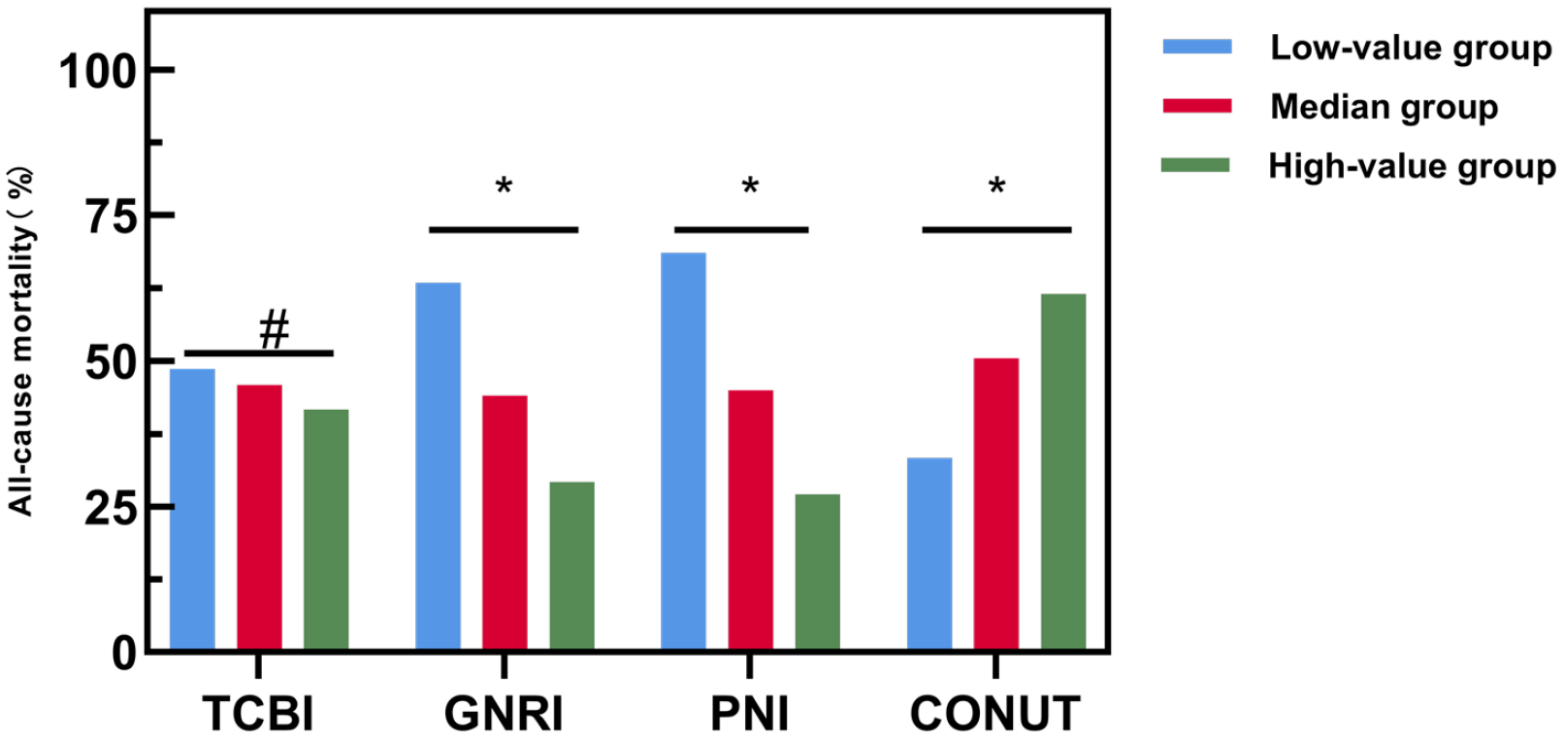
**Comparison of all-cause mortality among patients in different nutritional index groups.** Abbreviation: TCBI, Triglyceride-Total Cholesterol-Body Weight Index; GNRI, Geriatric Nutritional Risk Index; PNI, Prognostic Nutritional Index; CONUT, Controlled Nutrition Score. An asterisk (*) denotes a significance level of *p* < 0.05 in the comparison among three groups, while a hash symbol (#) indicates *p* > 0.05 for the same comparison.

The mean follow-up time was 59.02 ± 1.79 months, and during this time 99 (45.41%) elderly patients with HFpEF succumbed to all causes. A comparison between the all-cause mortality and survival groups across various nutritional indicators yielded the following insights (Figs. 2,3): (1) The distribution of patients in the all-cause mortality group across TCBI categories showed 35.4% in the low TCBI group, 34.3% in the median TCBI group, and 30.3% in the high TCBI group. While there was a trend towards an increase in the all-cause mortality rate in the low TCBI group compared to the other two groups, we did not observe any statistically significant differences among the three groups (*p* > 0.05). (2) Regarding the GNRI, 45.5% of the all-cause mortality fell into the low GNRI group, 33.3% were in the median GNRI group, and 21.2% were in the high GNRI group. Notably, the all-cause mortality rate was significantly higher in the low GNRI group compared to the median and high GNRI groups, exhibiting a statistically significant difference among the three groups (*p* < 0.05). (3) Examining the PNI categories within the all-cause mortality group, 50.5% were classified in the low PNI group, 30.3% in the median PNI group, and 19.2% in the high PNI group. Similar to GNRI, the all-cause mortality rate in the low PNI group was higher than both the median and high PNI groups, with a statistically significant difference among the three groups (*p* < 0.05). (4) Analysis of CONUT revealed that, 27.3% of the all-cause mortality cases were in the low CONUT group, 56.6% were in the median CONUT group, and 16.2% were in the high CONUT group. Remarkably, the all-cause mortality rate of the high CONUT group exceeded that of the of the median CONUT and low CONUT groups, with a statistically significant difference among the three groups (*p* < 0.05).

### 3.3 Survival Differences in Elderly HFpEF Patients across Differeing TCBI, GNRI, PNI, and CONUT Levels

**Fig. 4.**
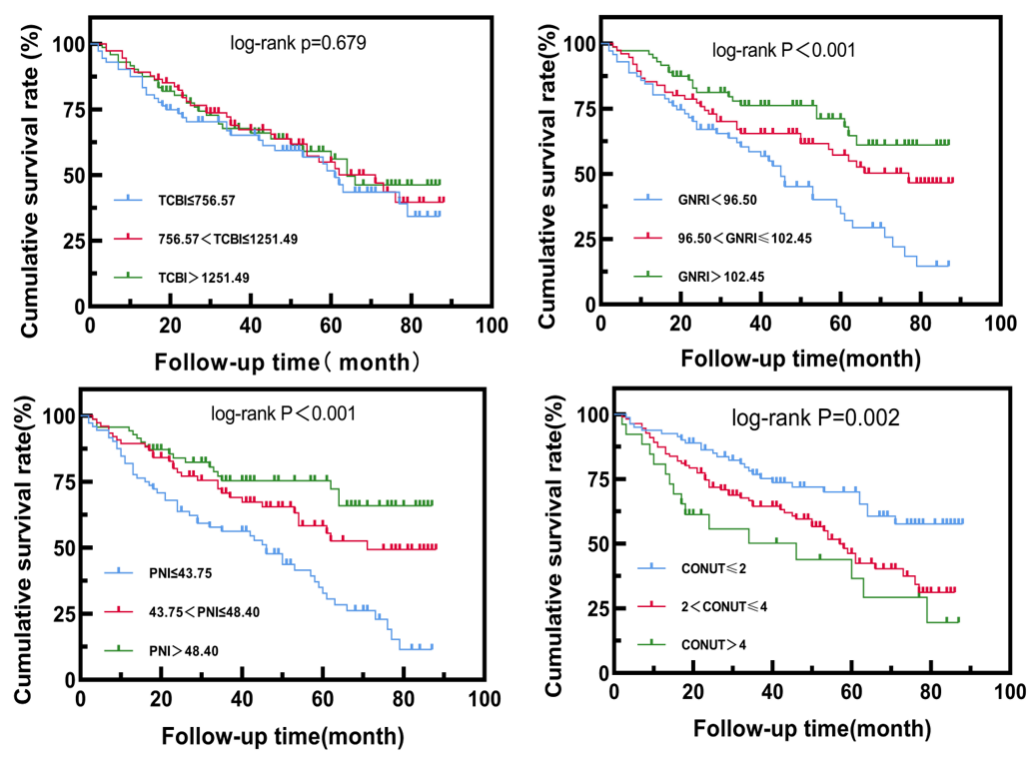
**Kaplan-Meier survival curve analysis of patients with different TCBI, GNRI, PNI, and CONUT subgroups.** Abbreviations: TCBI, Triglyceride-Total Cholesterol-Body Weight Index; GNRI, Geriatric Nutritional Risk Index; PNI, Prognostic Nutritional Index; CONUT, Controlled Nutrition Score.

The mean survival times of the three groups with low TCBI (≤756.57), intermediate (756.57–1251.49), and high (>1251.49) were 54.40 ± 3.96, 58.49 ± 3.73, and 58.40 ± 3.92 months, respectively. There was no statistically significant difference in the cumulative survival rate among the three groups (*p* > 0.05), as illustrated in Fig. 4.

The mean survival times for the three groups based on GNRI, categorized as low (≤96.50), medium (96.50–102.45), and high (>102.45), were 45.90 ± 3.59, 58.86 ± 3.88 and 66.83 ± 3.65 months, respectively. The cumulative survival rate of the GNRI medium and high groups increased in comparison with that of the GNRI low group (log-rank $X^{2}$ = 6.675, *p* = 0.010, log-rank $X^{2}$ = 15.84, *p* < 0.001). However, there was no statistically significant difference in the cumulative survival rate between the GNRI medium-value group and the high-value group (log-rank $X^{2}$= 2.432, *p* = 0.119), as depicted in Fig. 4.

The mean survival times for the three groups based on PNI, classified as the low-value group (≤43.75), mid-value group (43.75–48.40), and high-value group (>48.40), were 44.73 ± 3.46, 60.30 ± 3.87, and 68.21 ± 3.68 months, respectively. The cumulative survival rate of the PNI mid-value and high-value groups increased compared to that of the PNI low-value group (log-rank $X^{2}$ = 9.58, *p* = 0.002; log-rank $X^{2}$ = 20.32, *p* < 0.001). However, there was no statistically significant difference in cumulative survival between the PNI mid-value group and the high-value group (log-rank $X^{2}$ = 2.401, *p* = 0.121), as depicted in Fig. 4.

The mean survival times for the CONUT groups, categorized as low-value group (≤2), medium-value group (2–4), and high-value group (>4), were 66.28 ± 3.44 months, 53.07 ± 3.04 months, and 43.98 ± 6.68 months, respectively. The cumulative survival rate of the CONUT medium value group and the high-value group decreased compared to that of the CONUT low-value group (log-rank $X^{2}$ = 7.16, *p* = 0.007; log-rank $X^{2}$ = 11.09, *p* < 0.001). However, there was no statistically significant difference in cumulative survival between the CONUT medium-value group and the high-value group (log-rank $X^{2}$ = 1.847, *p* = 0.174), as shown in Fig. 4.

### 3.4 Multifactorial COX Proportional Risk Regression Modeling of All-Cause Mortality

The one-way COX regression analysis identified specific nutritional indices as risk factors for all-cause mortality in elderly HFpEF patients. Specifically, GNRI ≤96.50, PNI ≤43.75, and CONUT ≤2 were all associated with increased mortality risk (*p* < 0.05). In a more comprehensive analysis (Model 2), survival was the dependent variable (Assignment: 1 = Death, 0 = Survival). This model incorporated several independent variables: age (real value), sex (1 = Male, 0 = Female), BMI (real value), e GFR (real value), CCR (real value), BUN (real value), and the presence of chronic kidney disease (1 = Yes, 0 = No). The results from the multifactorial COX regression analysis indicated that among the nutritional indices, only a PNI ≤43.75 was found to be an independent risk factor for all-cause mortality in elderly patients with HFpEF (*p* < 0.05), as illustrated in Table 2.

**Table 2.** **Univariate and multivariate COX regression risk analysis of all-cause mortality in patients with HFpEF**.

Variation		Model 1			Model 2		
		HR (95% CI)	β-value	*p*-value	HR (95% CI)	β-value	*p*-value
TCBI							
	≤756.57	1.221 (0.750–1.989)	0.200	0.422	0.829 (0.490–1.402)	–0.188	0.483
	756.57 < TCBI ≤ 1251.49	1.035 (0.633–1.692)	0.035	0.890	1.053 (0.631–1.760)	0.052	0.842
	>1251.49	reference			reference		
GNRI							
	≤96.50	2.711 (1.613–4.556)	0.997	0.001	1.206 (0.659–2.208)	0.187	0.544
	96.50 < GNRI ≤ 102.45	1.517 (0.877–2.623)	0.417	0.136	1.257 (0.712–2.221)	0.229	0.431
	>102.45	reference			reference		
PNI							
	≤43.75	3.116 (1.835–5.292)	1.137	0.001	1.821 (1.039–3.193)	0.599	0.036
	43.75 < PNI ≤ 48.40	1.559 (0.877–2.770)	0.444	0.130	1.083 (0.591–1.985)	0.080	0.797
	>48.40	reference			reference		
CONUT							
	≤2	0.362 (0.195–0.674)	–1.016	0.001	0.564 (0.292–1.088)	–0.573	0.088
	2 < CONUT ≤ 4	0.670 (0.384–1.170)	–0.401	0.159	0.852 (0.474–1.530)	–0.160	0.591
	>4	reference			reference		

Abbreviations: TCBI, Triglyceride-Total Cholesterol-Body Weight Index; GNRI, Geriatric Nutritional Risk Index; PNI, Prognostic Nutritional Index; CONUT, Controlled Nutrition Score; HR, hazard ratio; CI, confidence interval; HFpEF, heart failure with preserved ejection fraction.

### 3.5 Comparison of the Predictive Value of TCBI, GNRI, PNI, and COUNT for All-Cause Mortality in Elderly Patients with HFpEF

**Fig. 5.**
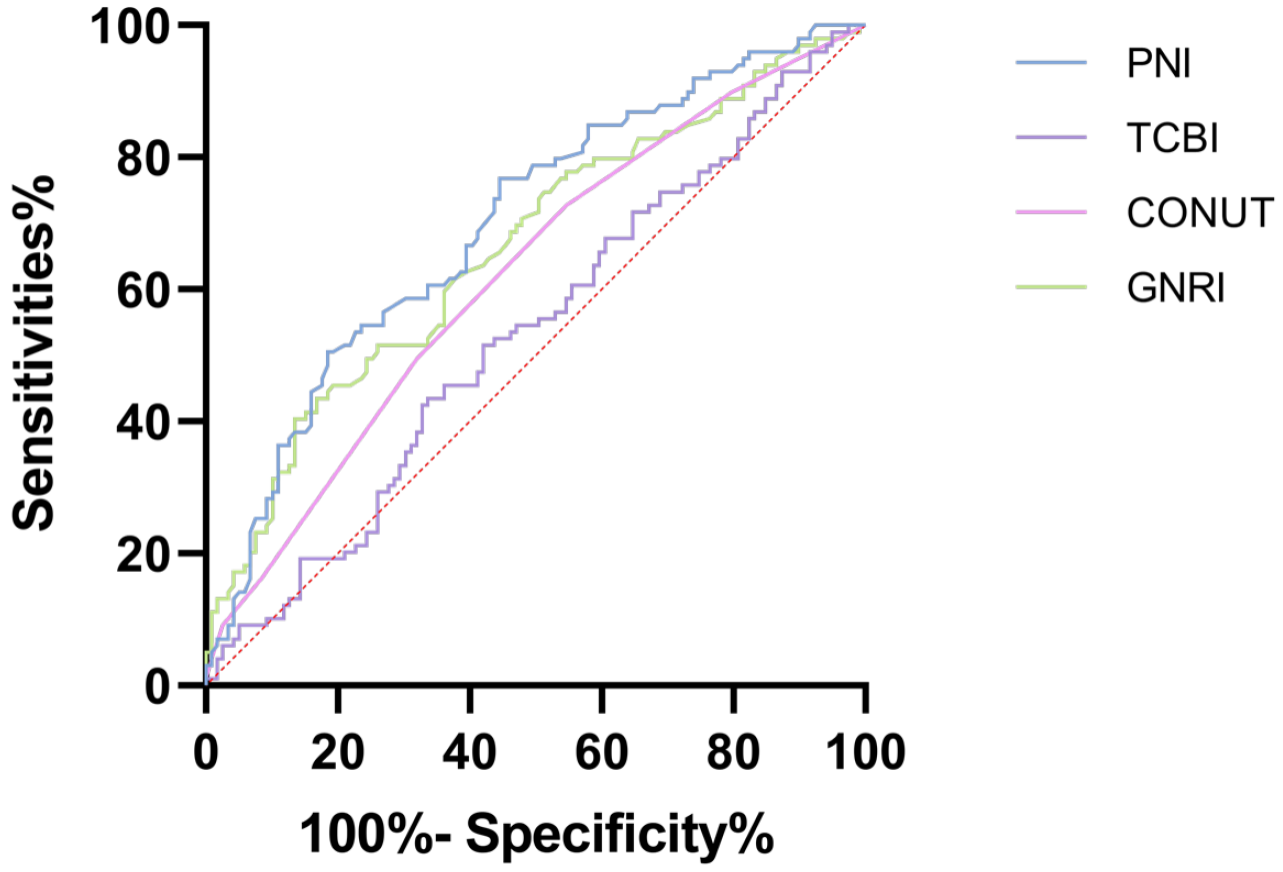
**ROC curves of TCBI, GNRI, PNI, and CONUT on the prognosis of HFpEF in older adults.** Abbreviations: TCBI, Triglyceride-Total Cholesterol-Body Weight Index; GNRI, Geriatric Nutritional Risk Index; PNI, Prognostic Nutritional Index; CONUT, Controlled Nutrition Score; HFpEF, heart failure with preserved ejection fraction; ROC, receiver operating characteristic.

In predicting all-cause mortality among elderly patients with HFpEF, the receiver operating characteristic (ROC) curves and Delong’s test were used to evaluate the performance of various nutritional indices including TCBI, PNI, GNRI, and CONUT. The predictive accuracy of PNI (AUC = 0.698, 95% CI 0.629–0.768) was superior to both TCBI (AUC = 0.533, 95% CI 0.456–0.610) and CONUT (AUC = 0.621, 95% CI 0.547–0.695), showing a statistically significant difference (*p* < 0.05). However, there was no statistically significant difference (*p* > 0.05) when compared with GNRI (AUC = 0.663, 95% CI 0.590–0.735), as shown in Table 3 and Fig. 5.

**Table 3.** **Clinical diagnostic value of TCBI, GNRI, PNI, and CONUT in predicting all-cause mortality in older adults with HFpEF**.

Variation	AUC	95% CI	Sensitivities	Specific	*p*-value	vs. TCBI	vs. GNRI	vs. PNI	vs. CONUT
TCBI	0.533	0.456–0.610	0.434	0.664	0.406	reference	0.005	0.001	0.035
GNRI	0.663	0.590–0.735	0.404	0.866	0.001	0.005	reference	0.185	0.350
PNI	0.698	0.629–0.768	0.768	0.555	0.001	0.001	0.185	reference	0.011
CONUT	0.621	0.547–0.695	0.727	0.454	0.002	0.035	0.350	0.011	reference

Abbreviations: TCBI, Triglyceride-Total Cholesterol-Body Weight Index; GNRI, Geriatric Nutritional Risk Index; PNI, Prognostic Nutritional Index; CONUT, Controlled Nutrition Score; HFpEF, heart failure with preserved ejection fraction; AUC, area under the curve.

## 4. Discussion

Malnutrition is strongly associated with the occurrence of numerous adverse outcomes such as increased mortality [10] and prolonged hospitalization [11]. Regular nutritional screening is recommended for older adults, with individuals aged 65 and above in the community being screened at least once every 6 months, and once every 3 months for those residing in nursing homes. In patients with heart failure, persistent low-grade inflammation can act on nociceptors, leading to skeletal muscle catabolism, delayed gastric emptying, and impaired release of appetite-controlling hormones. This often leads to a loss of appetite and an increased risk of malnutrition. Studies have demonstrated that the prevalence of malnutrition in this population can be as high as 69%, irrespective of age, sex, or left ventricular ejection fraction [12]. It is much higher than the 25% malnutrition risk [13, 14] of the elderly aged 65 and over in the community and nursing homes. The 2019 heart failure society of America (HFSA) [15] consensus statement on nutrition, obesity, and cachexia in patients with heart failure emphasizes that certain patients meeting the following criteria should undergo routine screening for malnutrition risk every 4–12 weeks. These criteria include: (1) BMI <20 or ≥30 kg/$m^{2}$ or unintentional, dry weight loss >7.5% within 6–12 months; (2) patients being considered for heart transplantation or mechanical circulatory support; (3) patients with heart failure who have been hospitalized for ≥7 days.

Despite these guidelines, there is no universally accepted methodology for nutritional assessment and screening of patients with heart failure. The single biochemical nutritional indexes, represented by BMI, PA, TG, etc., are susceptible to interference from various factors and cannot comprehensively assess the overall nutritional status of patients. In recent years, although numerous nutritional screening scale tools have also been developed, such as the nutritional risk screening score 2002 (NRS), the malnutrition universal screening tool (MUST), and the subjective globally assessment (SGA), the assessment items are cumbersome and subjective, and patients are required to have a certain degree of cognitive ability, which limits their clinical application. Therefore, to improve the long-term prognosis of heart failure patients, it is particularly important to screen simple, efficient, and comprehensive nutritional assessment indexes.

The results of our study indicate that the cumulative survival rate was significantly lower in the low PNI group compared to both the median and high PNI group (log-rank $X^{2}$ = 9.58, *p* = 0.002; log-rank $X^{2}$ = 20.32, *p* < 0.001). Additionally, the low PNI group was identified as an independent risk factor for all-cause mortality in patients with HFpEF. The PNI is a readily available and noninvasive comprehensive nutritional assessment tool. Low PNI levels typically indicate a relative decrease in albumin or lymphocyte counts. A decrease in albumin can lead to pulmonary and myocardial edema, fluid retention, diuretic resistance, oxidative stress, and an exacerbated inflammatory response, all contributing to disease progression in patients with HF. Ming Liu *et al*. [16] identified low albumin as an independent risk factor for 1-year all-cause mortality in HFpEF patients. Similarly, lymphocyte count is an important prognostic factor [17]. Heart failure can trigger the release of endotoxins, causing lymphocyte apoptosis and infiltration of activated T cells in cardiac tissues, leading to a decrease in lymphocyte count. A low lymphocyte count indicates reduced immune system activity, rendering the patient more susceptible to pathogens. In conclusion, PNI is a valuable nutritional indicator for predicting all-cause mortality in HFpEF patients. Future nutritional interventional studies can be designed to assess various factors based on the components of this indicator.

This study also revealed that PNI (AUC = 0.698, 95% CI 0.629–0.768) outperformed both TCBI (AUC = 0.533, 95% CI 0.456–0.610) and CONUT (AUC = 0.621, 95% CI 0.547–0.695) in predicting all-cause mortality in patients with HFpEF (*p* < 0.05). There are several reasons why PNI is a more effective predictor. Unlike the CONUT score, PNI is calculated using albumin and lymphocyte counts as continuous variables, reducing information loss and more accurately reflecting the nutritional status. At the same time, albumin and lymphocyte count are also closely related to the occurrence of sarcopenia, a common complication caused by malnutrition. In 2010, the European working group on sarcopenia in older people (EWGSOP) [18] defined sarcopenia as a common geriatric syndrome characterized by progressive and generalized loss of skeletal muscle mass and strength. It was recognized that ${\mathrm{CD8}}^{+}$ T lymphocytes can penetrate damaged muscle, promote the secretion of monocyte chemoattractant protein-1 (MCP-1), recruit Gr1 macrophages, and participate in muscle cell proliferation [19].

During immune aging, the decrease and alteration of T lymphocyte phenotype (from ${\mathrm{CD8}}^{+}$ to ${\mathrm{CD4}}^{+}$) are also related to the decrease of muscle mass [20]. Albumin is a suitable biomarker to reflect the state of human visceral protein [21]. Studies have shown that plasma albumin levels are higher in elderly individuals without sarcopenia compared to those with sarcopenia [21]. This discrepancy may result from low albumin indicating reduced protein storage, subsequently stimulating catabolic processes leading to muscle breakdown. In addition, albumin serves as a specific regulator of cellular glutathione, a critical antioxidant [22]. Thus, glutathione plays a crucial role in mitigating oxidative stress, which is particularly important to the age-related decline of skeletal muscle [23]. PNI, by integrating the sarcopenia diagnosis and medical nutritional management, proves more effective in the prognostic assessment of patients with heart failure with preserved ejection fraction. Additionally, lymphocyte counts are a more stable long-term indicator of body composition compared to calculated indicators like GNRI and TCBI (body weight, TC, TG) which are susceptible to influences such as age, diet, medication, and other lifestyle habits. Despite obtaining this biochemical index after a 24-hour fasting period, their short-term variability should be considered. These variations, despite standardized fasting, can impact their accuracy in predicting patient outcomes.

Recent years have seen a growing use of admission indices like CONUT, GNRI, and TCBI for prognostic prediction in cardiovascular diseases such as transcatheter aortic replacement [24] and acute myocardial infarction [25]. For example, Xin Deng* et al*. [26] investigated patients with ST-segment elevation acute myocardial infarction who underwent percutaneous coronary intervention (PCI). These patients were categorized based on their CONUT score into high (≥5), middle (2–4), and low (≤1) CONUT groups [26]. The study found that the high CONUT group experienced a significantly higher incidence of major adverse cardiovascular events (MACE), myocardial reinfarction, and vascular revascularization compared to the middle and low CONUT groups [26]. After adjusting for age, sex, left ventricular ejection fraction, and creatinine in a multifactorial COX regression analysis, a high CONUT score remained an independent risk factor for MACE (hazard ratio [HR]: 12.09, 95% confidence interval [CI]: 5.09–28.7, *p* < 0.001). Wang *et al*. [27] identified the TCBI low-value group (<701) as an independent risk factor for all-cause mortality in patients with dilated cardiomyopathy. However, there is a limited number of studies investigating the relationship between CONUT, GNRI, TCBI, and HFpEF prognosis both domestically and internationally.

The results of the present study revealed that the overall survival rate was lower in the low GNRI group, the low TCBI group, and the high CONUT group compared to their respective high GNRI, high TCBI, and low CONUT counterparts. Nevertheless, further multifactorial COX regression analysis demonstrated that GNRI, CONUT, and TCBI were not independent risk factors for all-cause mortality in HFpEF. This outcome may be attributed to the characteristics of the population, predominantly elderly heart failure patients with an average age around 85 (77, 89) years. Notably, 23.85% (52) of these patients were in the decompensated stage of HFpEF and had a high usage rate of intravenous diuretics (34.40%). The extensive use of diuretics, often leading to high fluid retention, might have resulted in increased body weights, potentially obscuring the loss of fat-free body mass and leading to an overestimation of their nutritional status. Furthermore, the majority of patients were on statins (45.4%), which lower TC and TG but contribute to stabilizing atherosclerotic plaques and extending survival cycles. Due to these factors, the study concluded that TCBI, GNRI, and CONUT may not be effective for prognostic assessment in elderly HFpEF patients, as their results could be skewed by the high-water load and the effects of medications like statins.

### Study Limitations

This study has several limitations. Firstly, it is a single-center, retrospective cohort study with a small sample size, necessitating validation through large-scale prospective and multi-center studies in the future. Secondly, the assessment of nutritional status in patients with heart failure with preserved ejection fraction was limited to a single point in time, specifically upon their first admission. This approach neglects the potential benefits of a dynamic assessment of nutritional status, both during hospitalization and after discharge. Lastly, the investigation focused solely on the association of nutritional status with all-cause mortality. Other outcomes, such as cause-specific mortality (e.g., cardiovascular mortality, cancer mortality), and readmission outcomes were not explored. This narrow focus may overlook other important aspects of patient health and outcomes related to HFpEF.

## 5. Conclusions

In summary, low PNI is an independent prognostic risk factor for patients with HFpEF, demonstrating a higher prognostic diagnostic value compared to TCBI, GNRI, and CONUT.

## Data Availability

All data generated or analyzed during this study are included in this published article or are available from the corresponding author on reasonable request.
